# Computational Discovery of Design Principles for Plasmon-Driven Bond Activation on Alloy Antenna Reactors

**DOI:** 10.1021/acsnano.4c13602

**Published:** 2025-03-07

**Authors:** Connor J. Herring, Matthew M. Montemore

**Affiliations:** Department of Chemical and Biomolecular Engineering, Tulane University, New Orleans, Louisiana 70115, United States

**Keywords:** plasmonic catalysis, RT-TDDFT, photocatalysis, methane activation, CO_2_ activation, water activation, N_2_ activation

## Abstract

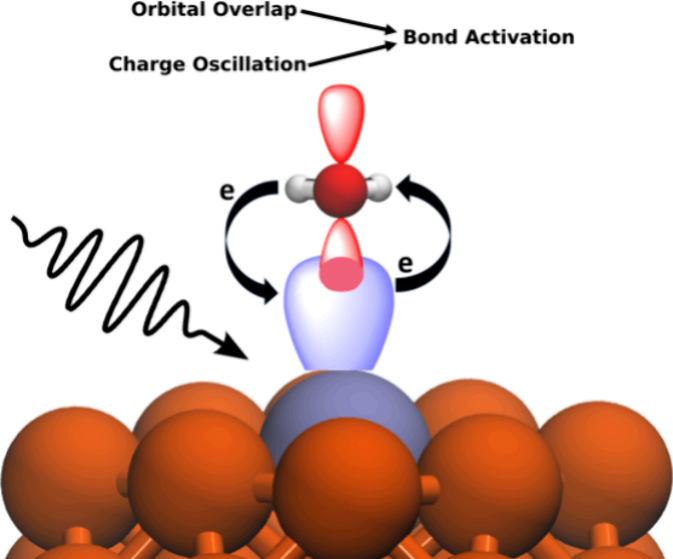

Plasmonic “antenna reactor” alloys, consisting of a plasmonic material doped with a catalytically active metal, show great promise for efficient photocatalysis. However, while simple, intuitive, and approximate design principles such as the Sabatier principle have been developed for thermal and electrocatalysis, similar design principles for plasmonic catalysts remain elusive. Here, we develop these simple design principles by using real-time, time-dependent density functional theory to study small molecule activation (CH_4_, CO_2_, H_2_O, and N_2_) on a number of Cu-based antenna reactors and elucidate trends. We first show that this technique gives results consistent with experimental plasmonic catalysis studies. We then identify promising, previously untested antenna reactors for these molecules. Next, we find that, for a given molecule, bond activation correlates with the size of the charge oscillations between the nanoparticle and molecule as quantified by the standard deviation over the propagation time. Furthermore, the orbital overlap between the dopant and molecule also roughly correlates with the bond activation. For CH_4_, N_2_, and H_2_O, a greater overlap leads to higher activation. For CO_2_, the trend is reversed because a greater overlap leads to higher chemical activation upon adsorption, which inhibits photoactivation. Hence, the orbital overlap can be used as a computationally efficient and intuitively simple predictor of photoactivation for the initial screening of plasmonic catalysts.

## Introduction

Plasmonic photocatalysts have shown great promise for utilizing light to drive chemical reactions with high selectivity and at mild conditions.^1−3^ These factors, among others, may make them more economically and environmentally suitable than thermal catalysts for some technologies. The antenna-reactor architecture, particularly alloys, allows for fine-tuning the system properties for high performance. In this design scheme, an optically active material (the antenna) can drive excitation in an adjacent or alloyed metal which is poorly light-absorbing (the reactor).^4^ Cu as a host metal has particular advantages such as a lower cost compared to Au and Ag.^5^ However, the lack of simple, concrete design principles makes improving plasmonic catalysts difficult. Here, we develop these design principles for activating strong bonds using dynamic, nonadiabatic simulations based on real-time time-dependent density functional theory (RT-TDDFT).

We focus on small molecules which are both hard to activate and involved in technologically important catalytic reactions such as CO_2_ and CH_4_ conversion, H_2_ production from H_2_O, and ammonia synthesis. Generally, an ideal catalyst for these types of reactions can activate the relatively inert reactants but maintain weak adsorption for downstream reaction intermediates. However, this can be difficult to accomplish using traditional catalysts as there is often a trade-off between these factors wherein facilitating reactant activation results in strong binding, potentially poisoning the catalyst. This trade-off, known as the Sabatier principle, limits overall catalytic performance. Plasmonic photocatalysts may circumvent this trade-off by using light to facilitate reactant activation without strengthening adsorption, which is particularly important for molecules that are difficult to activate.

Simple principles for understanding trends in reactivity and catalytic performance, such as the aforementioned Sabatier principle or the d-band model, have played a crucial role in catalysis science.^6,7^ These types of simple pictures are approximate, and often cannot make predictions with high quantitative accuracy;^6,8,9^ however, they are quite powerful for initial screening, building intuition, and rationalizing trends. However, analogous principles for plasmonic catalysis have not been developed. Thus, it is important to establish such predictive design principles to aid in efficient screening and design as well as fundamental understanding of trends.

RT-TDDFT is a powerful tool for studying ultrafast, excited-state dynamics such as those present in plasmonic excitations. To this end, it has been used to study a wide range of plasmonic systems including nanoparticles and clusters,^10−12^ linear chains of atoms,^13−16^ and many others.^17^ While RT-TDDFT has been relatively widely applied, it has not yet been used to investigate plasmonic antenna reactor nanoparticles or to develop simple descriptors for predicting photocatalytic activity. As noted below, we find that RT-TDDFT reproduces known experimental trends for antenna reactors.

After validating our methodology by comparison to experiment, we searched for simple descriptors for plasmon-driven bond activation by performing a series of RT-TDDFT calculations with N_2_, H_2_O, CH_4_, and CO_2_ adsorbed on five different Cu-based antenna reactors (doped with Ag, Mo, Re, Ru, or Ti), as well as pure Cu. Our results showed that charge transfer to the molecule—largely in the form of oscillations and captured by the charge’s standard deviation over the propagation time—correlated with the change in bond length. However, while this provides rationalization of trends, it also requires running a computationally expensive RT-TDDFT calculation and is thus challenging to use for screening. Thus, we also tested the utility of the orbital overlap matrix to predict bond activation, and found that the maximum orbital overlap value between the adsorbate and dopant can reasonably predict bond activation. Since the overlap matrix is easy to calculate from a simple relaxation, this could greatly accelerate the screening process—as well as providing improved fundamental understanding of trends—for plasmonic antenna reactors.

## Results and Discussion

First, we tested whether our RT-TDDFT methodology can correctly reproduce trends from experimental studies of plasmonic antenna reactors by performing calculations for comparisons made in four previous experimental studies. These studies included dry reforming of methane (DRM) on single-site RuCu antenna reactors as compared to pure Cu,^18^ DRM on PtAu nanostructures as compared to pure Au,^19^ nitrogen fixation on Pd nano-octahedron/Ru arrays as compared to pure Ru,^20^ and methane steam reforming (MSR) on RhCu antenna reactors as compared to pure Cu.^21^ For each case, we studied the step that is thought to be rate-determining: CH_4_ activation for DRM and MSR, and N_2_ activation for N_2_ fixation. After relaxing each system, we subjected it to an oscillating electric field to mimic light (see the Methods section) and then tracked bond activation in the molecules. We then compared whether the nanomaterial that gave higher bond activation in our simulations also gave higher experimental catalytic activity, and found that our computational studies gave the correct trend in all cases. That is, our RT-TDDFT simulations showed a significant enhancement in bond activation for the antenna reactor complexes as compared to the pure-metal systems (see Figures S12–S14). The qualitative results are shown in Table 1 and more detail is given in Section S7.

**Table 1:** Comparison between Experimental and RT-TDDFT Simulated Antenna Reactors^a^

**Reaction**	**materials compared**	**experimentally more active**	**computationally more active**	**ref**
DRM	Cu vs Ru–Cu alloy	Ru–Cu > Cu	Ru–Cu > Cu	(18)
DRM	Au vs Pt–Au nanostructure	Pt–Au > Au	Pt–Au > Au	(19)
N_2_ fixation	Ru vs Ru–Pd nanostructure	Ru–Pd > Ru	Ru–Pd > Ru	(20)
MSR	Cu vs Rh–Cu alloy	Rh–Cu > Cu	Rh–Cu > Cu	(21)

a RT-TDDFT simulations show complete agreement with the experiment in predicting photocatalytic activity trends.

**Figure 1:**
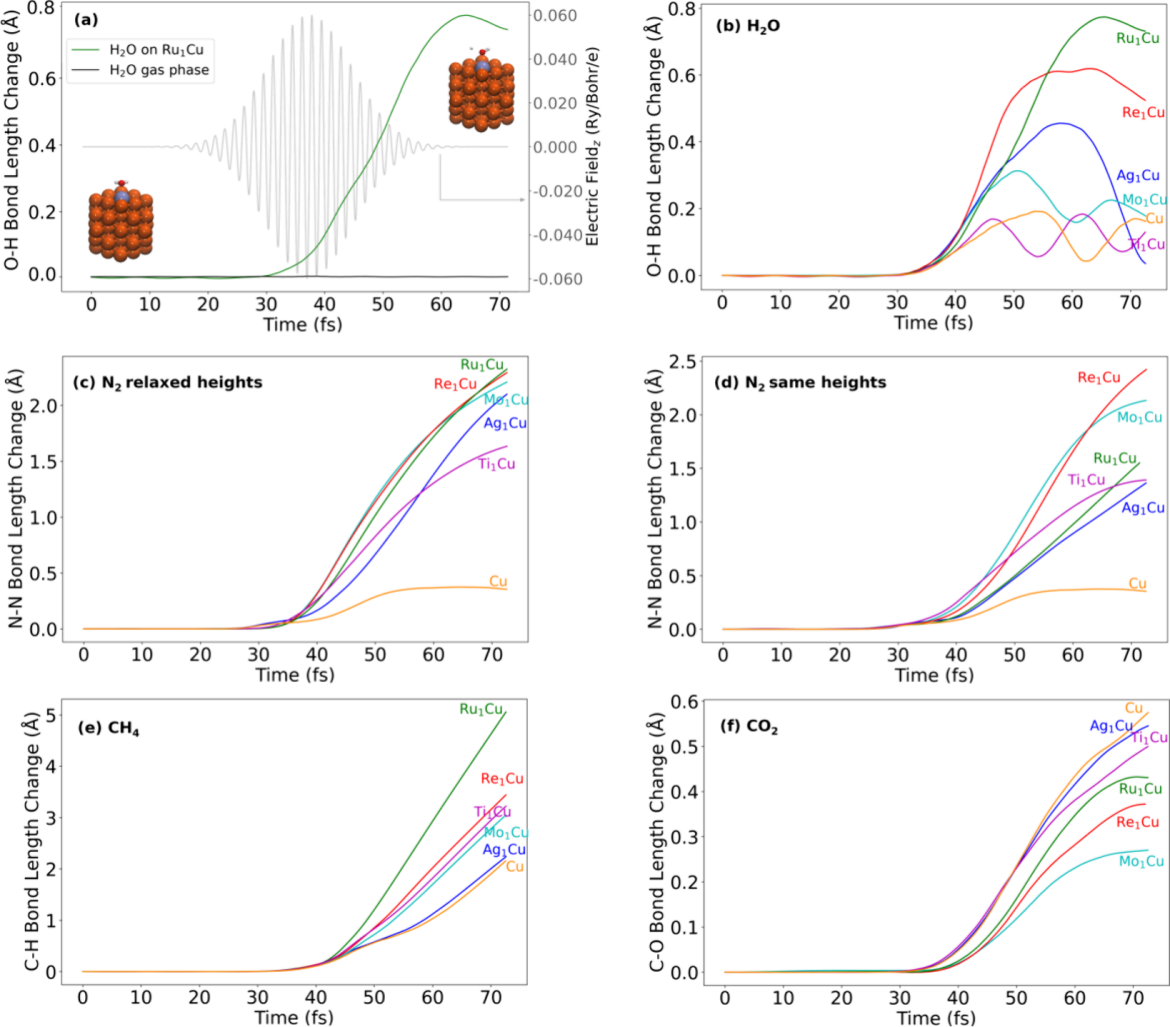
Trends in photoactivation depend on the molecule. (a) Simulation of H_2_O subjected to an oscillating electric field representing light exposure (with this field shown on the right axis), both in the gas phase and on a Ru_1_Cu nanoparticle, with the initial and final geometries on the nanoparticle. The bond length change for the most-activated O–H bond is shown over time. (b–f) Bond length change vs time for the most activated bond in H_2_O, N_2_, CH_4_, and CO_2_ on all nanoparticles.

We next performed calculations of N_2_, H_2_O, CH_4_, and CO_2_ adsorbed on a set of alloy antenna-reactor nanoparticles and exposed them to light, finding that the nanoparticles all facilitated photoactivation but to varying degrees. For example, light exposure significantly activated O–H bonds for H_2_O adsorbed on all of the nanoparticles, even though the same field had a negligible effect on H_2_O in the gas phase (see Figure 1a). In this case, the Ru_1_Cu alloy gave the highest level of activation, increasing one O–H bond by more than 0.7 Å (relative to the relaxed O–H bond at the start of the simulation). The nanoparticles’ photoactivity trends varied significantly between molecules (Figure 1b–f). However, one commonality was that the Ru_1_Cu nanoparticle generally showed substantial activation for all molecules and was the most activating nanoparticle except for CO_2_. This is broadly consistent with previous studies showing that Ru-doped plasmonic metals can be effective photocatalysts.^22,23,18,24^ A simple test of thermal effects suggests that thermalizing the systems does not alter trends (see Figure S17). For all of the molecules except for CO_2_ (discussed below), most alloyed structures showed an increased level of photoactivation relative to pure Cu. Thus, our results qualitatively captured the notion that antenna-reactor structures often show improved catalytic activity compared to their pure-metal counterparts.^18,22,24^ These results also show that the propensity for photoactivation is not solely a function of the nanoparticle itself or its properties, as the significant variations in trends for different molecules show that some feature(s) of the nanoparticle-molecule interaction is critical in determining photoactivation. We also note that due to the strong applied field in RT-TDDFT studies of photochemistry, the dynamics are not a realistic approximation of the behavior of most experimental systems over the same time scale. Instead, we find that the trends in photo activation across systems are in agreement with experimental trends. In general, we do not extract quantitative values but instead the ordering of which systems give most photoactivation.

One possible effect of the dopant is to move the adsorbing species closer or farther from the nanoparticle, affecting the strength of nanoparticle-molecule electronic coupling and the local field intensity. Since the calculations discussed thus far (Figure 1a–c,e,f) were all performed using the relaxed geometry as the initial state, the adsorbate-nanoparticle distance varies. To disentangle this effect, we performed a set of calculations for N_2_ at a consistent height above all of the nanoparticles. For these calculations, the pure Cu nanoparticle was chosen as the control height since it featured the largest N_2_-surface distance (3.09 Å). The consistent-height calculations revealed that the adsorbate height had only a moderate effect on the trends. At the relaxed heights the Ru, Re, and Mo alloys exhibited the largest N_2_ bond activation, followed by Ti, Ag and Cu. When moving the N_2_ to the same height, the degree of dissociation was reduced on the Ag and Ru alloys but relatively unchanged on the Mo and Ti alloys as well as pure Cu. The Re alloy showed a slight enhancement relative to its relaxed height, which may be due to strong adsorption in the relaxed geometry, similar to the effect discussed for CO_2_ below. Thus, while the dopant’s effect on the adsorption height had a non-negligible effect on photoactivation, other system properties appear to be much more important in driving trends.

Adsorption energies were next considered as a possible metric for predicting a molecule’s degree of photoactivation, where photoactivation was measured by the change in bond length induced by the external field (see Figure S1). While there is some correlation between these two parameters for some molecules, with stronger adsorption roughly corresponding to higher activation for N_2_ and CH_4_, this relationship is neither accurate nor generally applicable. For example, there is no apparent relationship between photoactivation and adsorption strength for H_2_O. Additionally, Ag_1_Cu and Cu bind N_2_ with similar strengths yet the N_2_ bond length change was very different: 2.1 Å on Ag_1_Cu and just 0.37 Å on Cu. Furthermore, Ru_1_Cu was the most effective for photoactivation of CH_4_ and H_2_O but bound these molecules only moderately compared to other nanoparticles. Lastly, the trend switches for CO_2_, as the more strongly bound cases led to less C=O bond activation. Hence, the trends observed in Figure 1 are not well explained by variations in adsorption strength.

**Figure 2:**
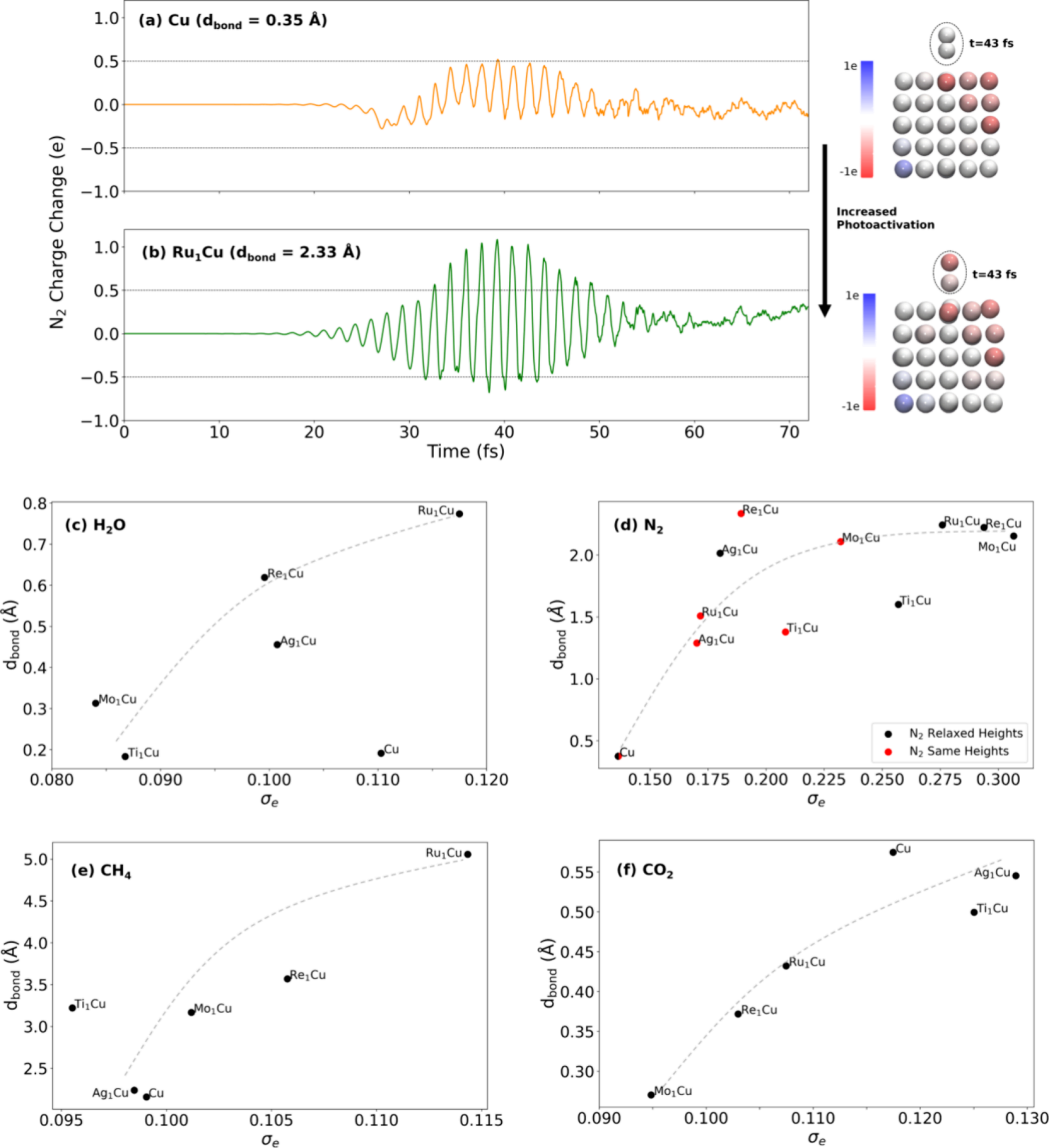
Standard deviation of charge transfer correlates with bond activation. (a, b) Charge on N_2_, relative to the initial charge, on (a) Ru_1_Cu and (b) pure Cu, with dashed lines at ±0.5 e. The N_2_ bond length change over the course of the simulation, *d*_bond_, is given. The geometries at a time of 43 fs are also shown, with every atom colored by its net charge and a dashed oval around N_2_. (c–f) Standard deviation in charge change, σ_e_, on the most-activated bond is plotted with *d*_bond_ for that bond: (c) H_2_O, (d) N_2_, (e) CH_4_, and (f) CO_2_. Lines have been added to guide the eye to the overall trends but are not lines of best fit.

To further investigate the underlying driver of trends in molecular dissociation we turned to charge transfer between the nanoparticle and molecule, as previous studies have suggested that charge transfer may be important.^17,25−28^ Hirshfeld charge partitioning^29^ was used to track the change in charge on each atom relative to the initial charge. We focused on the most highly activated bond for tracking both the change in bond distance and this change in charge. Examining the charge change for N_2_ on the least activating nanoparticle (Cu) and the most activating nanoparticle (Ru_1_Cu) (Figure 2; see Figure S7 for others) shows that the charge oscillations were much larger for Ru_1_Cu than for Cu. More generally, we found that systems with larger oscillations in charge transfer between the nanoparticle and N_2_ tended to feature a higher degree of photoactivation (i.e., a larger N_2_ bond length change from the initial state). The time average of the charge transfer is near zero in all of the N_2_ systems, indicating that electron transfer is primarily transient. Spatially, the charge that initially transferred to N_2_ mostly appeared on the N atom farther from the surface, as shown in the charge density difference calculations (Figure S5). To rule out the contribution of atomic motion to the change in charge, we also performed calculations with atomic positions fixed. In these cases, the charge oscillations closely resembled the unconstrained system and the structures which highly activated N_2_ in the unconstrained system again produced the largest charge oscillations when the atoms were fixed (Figure S3). Examining the other molecules revealed a similar correspondence between transient charge transfer and bond activation, although for these cases the trend is harder to see directly from plots of the charge difference (Figure S7).

**Figure 3:**
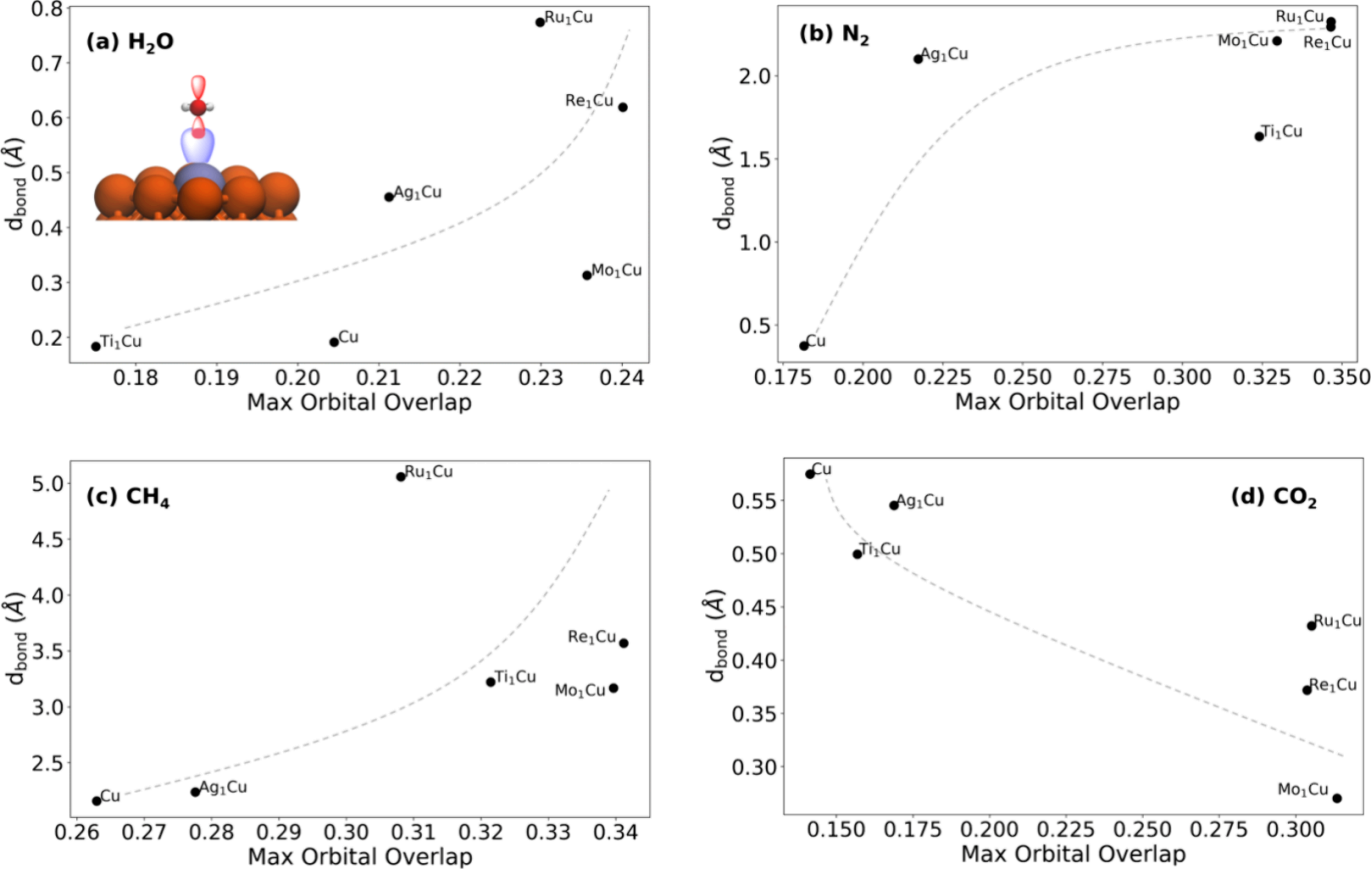
Orbital overlap is a simple descriptor for bond activation. (a–d) Degree of bond activation *d*_bond_ vs maximum overlap matrix value between the molecule and dopant atom. Lines have been added to guide the eye to the overall trends but are not lines of best fit. A schematic of the orbital overlap for H_2_O is shown in panel (a) as an example, but it does not necessarily reflect the actual orbitals involved in the maximum overlap.

To quantify the charge transfer oscillations between the molecule and nanoparticle in a simple way, the standard deviation of the charge change (σ_e_) for the atoms in the most activated bond was calculated. Plotting the bond length change *d*_bond_ against σ_e_ (Figure 3a–d) revealed a clear trend between charge standard deviation and bond activation. Namely, a larger standard deviation of charge transfer on a particular bond led to a greater degree of activation for that bond. There is a single large outlier, H_2_O/Cu, and there is generally some scatter in the plots, indicating that the standard deviation does not perfectly predict bond activation. Nevertheless, the clear and consistent trend across the molecules indicates that the standard deviation of charge transfer is a strong, qualitative indicator of bond activation.

While most of the systems featured charge oscillations about a mean of roughly zero over the entire simulation, a few cases exhibited a drifting mean, indicating nontransient charge transfer. Most notably, for CO_2_ on the Mo, Re, and Ru alloys, the oscillations in charge appear visually larger than the other three structures (see Figure S8), despite showing less bond activation. However, there is an overall decrease in charge for Ag_1_Cu, Ti_1_Cu, and Cu. This negative, nontransient charge transfer could also drive activation,^30^ and is also captured by the standard deviation. Hence, σ_e_ is robust to nontransient charge transfer (meaning a change in charge that persists at the end of the simulation time) in addition to the rapid, transient charge transfer.

While the standard deviation of charge transfer appears to explain photoactivation to a strong degree, it is not convenient to calculate nor easily intuitable in terms of design. Thus, to understand which system properties may lead to larger charge transfer and thus higher photoactivation, the orbital overlap between the molecule and the nanoparticle was also calculated. Specifically, the maximum overlap matrix value between the dopant and molecule was extracted for each case. We hypothesized that systems with higher orbital overlap between the molecule and dopant would tend to feature larger σ_e_ and higher photoactivation. (We also tested the use of the Hamiltonian matrix in place of the overlap matrix; this gave qualitatively similar results to the overlap matrix as shown in Figure S9.)

The orbital overlap matrix plots show that overlap between the adsorbate and dopant atom indeed generally correlates with the degree of bond activation, with CH_4_/Ru_1_Cu as the largest outlier. For H_2_O, N_2_, and CH_4_, more overlap corresponded to more bond activation. This is physically reasonable, as more overlap may tend to lead to stronger interactions and easier electron transfer. However, for CO_2_ the relationship between overlap and bond activation was reversed, prompting additional examination of these systems.

**Figure 4:**
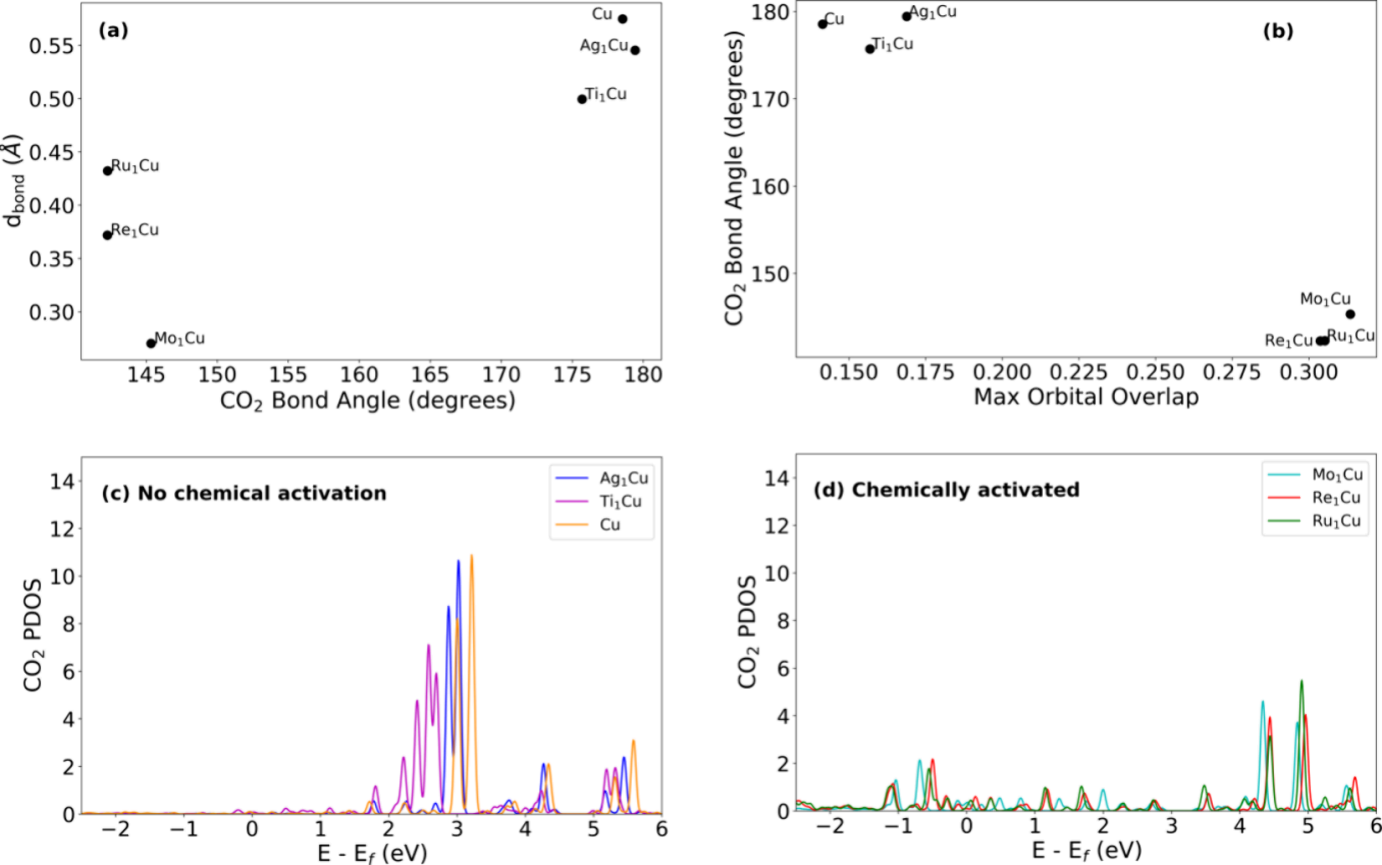
Chemical activation of CO_2_ inhibits photoactivation and correlates with the orbital overlap. (a) CO_2_ bond angle in the relaxed, initial geometry vs the maximum change in the C–O bond length upon light exposure. (b) Relaxed CO_2_ bond angle vs the maximum orbital overlap between the molecule and dopant. (c) CO_2_ PDOS for the nonchemically activated cases and (d) PDOS on CO_2_ for the chemically activating nanoparticles. Chemical activation leads to an upward shift in the CO_2_ states.

One possible reason for the trend reversal for CO_2_ is that it was chemically activated by some of the nanoparticles, which may influence the propensity for photoactivation. Specifically, we found that the Mo, Re, and Ru alloys, which showed the lowest photoactivation, all chemically activated the CO_2_, as evidenced by a decrease in the CO_2_ bond angle upon relaxation from roughly 180° to approximately 145° (Figure 4a), in agreement with previous work.^31^ To understand why chemical activation may inhibit photoactivation, we examined the projected density of states (PDOS) on CO_2_ in all cases, and found that chemical activation shifted the unoccupied CO_2_ molecular states to higher energies (Figure 4c,d). This will tend to make charge transfer more difficult, and indeed the level of charge transfer was reduced for the Mo, Re, and Ru alloys, as seen by the smaller σ_e_ values in Figure 3d. Thus, for CO_2_ it appears that increased overlap correlates with stronger chemical activation, and this chemical activation modifies the CO_2_’s electronic structure to make charge transfer more difficult, which impedes photoactivation. This additional effect of overlap apparently overrides the direct impact of overlap on photoactivation seen for the other molecules (Figure 3a–c), resulting in the reversed overall trend between overlap and photoactivation for CO_2_.

Hence, the orbital overlap can be a reasonable predictor of photocatalytic activity, and higher overlap leads to higher photoactivation in cases where there is not a significant difference in chemical activation between nanoparticles. When there are significant variations in the chemical activation and thus electronic structure of the adsorbed molecule, the overlap can correlate with those variations and thereby change the trend. As with other simple descriptors in catalysis, such as the hydrogen adsorption energy for hydrogen evolution,^9^ the orbital overlap is not a perfect predictor but rather an intuitive and computationally convenient guide for both screening and rationalization.

## Conclusions

In this work, RT-TDDFT has revealed trends and predictive design principles for the photochemical activation of CH_4_, CO_2_, H_2_O, and N_2_ on Cu-based antenna reactors. Our results showed that the doped structures often enhance the extent of bond activation compared to pure Cu. The exception to this trend is CO_2_, where differences in chemical activation sometimes disrupted the trends seen for other molecules. Further, these results identified Cu-based antenna reactors, especially RuCu and ReCu, with promising photoactivation properties for water splitting, as well as N_2_ and CH_4_ activation. We also showed that the standard deviation of the charge transfer (which is largely transient) over time correlates with bond activation, revealing the importance of charge exchange between the nanostructure and adsorbate states and providing an electronic-level explanation for trends in photoactivation. Lastly, the orbital overlap between the adsorbate and dopant was found to correspond reasonably well with bond activation. Higher overlap tends to result in higher activation for CH_4_, H_2_O, and N_2_, but for CO_2_ higher overlap also correlates with chemical activation which inhibits photoactivation. As the overlap can be obtained from relaxation calculations and gives a simple, intuitive explanation for trends, it may prove to be a useful method for rapidly screening many plasmonic structures for their ability to photoactivate a given reactant.

## Methods

Simulations were performed using RT-TDDFT with Ehrenfest dynamics—specifically, the computational package Time-evolving Deterministic Atom Propagator (TDAP).^32^ Nanoparticles were constructed using 55 atoms with the dopant located in the center of the (100) facet. The system was then relaxed after placing the adsorbate on the dopant atom, unless constraints were implemented as noted in the text. N_2_ was studied both in an end-on and side-on orientation; however, adsorption energies were always found to be lower in the end-on orientation, in agreement with several other computational studies,^33,34,31^ except for pure Cu, which displayed very similar energies in the two configurations (Table S1). Therefore, our calculations used N_2_ in the end-on geometry unless otherwise specified. Calculations were also run with N_2_ in the side-on orientation and in these cases the extent of dissociation was enhanced compared to the end-on configuration for all nanoparticles except Ag_1_Cu (see Figure S2).

Similar to previous work,^30,35−37^ light exposure was modeled using an oscillating electric field with a frequency of 2.5 eV. This field was applied to the system in all three directions using a Gaussian envelope with a width of approximately 50 fs. The field was initially zero and increased to its largest value at ∼40 fs before returning to zero once more by the end of the simulation (∼72 fs). For N_2_ the amplitude of the electric field was 0.1 Ry/Bohr/e (2.57 V/Å), but a weaker field (0.06 Ry/Bohr/e or 1.54 V/Å) was used for CH_4_, CO_2_, and H_2_O as the higher-intensity field was too strong to observe clear differences in activation for these three molecules. Since we are only interested in qualitative trends across nanoparticles for a given molecule, this computational setup is effective for our purposes. A time step of 0.5 ℏ/Ry, approximately 24 attoseconds, was used in all calculations. The frequency of 2.5 eV (496 nm) was chosen partly for consistency with our own previous work with Au and Ag nanoparticles of the same size. This wavelength corresponds to a blue-green color (near the peak of the solar spectrum) and falls within the LSPR peak region for many coinage metal nanoparticles.^5,30,38,39^

We also tested whether the degree of bond activation from these calculations correlates with a different measure of the propensity for photoactivation across nanoparticles. Specifically, for some of the nanoparticles (Mo_1_Cu, Ti_1_Cu, and pure Cu) the minimum electric field amplitude required to dissociate N_2_ was determined by varying the amplitude from 0.01 to greater than 0.1 Ry/Bohr/e (see Figure S6). This threshold field amplitude for Mo_1_Cu, Ti_1_Cu, and pure Cu was found via interpolation to be approximately 0.060, 0.074, 0.107 Ry/Bohr/e, respectively. This trend agrees with the findings from Figure 1d,e; that is, the more strongly activating alloys at a given field strength also require a lower amplitude electric field to dissociate N_2_. This supports the use of the maximum bond length achieved in the simulations in Figure 1 for capturing the trends in photoactivity.

All calculations were performed inside a 25 × 25 × 25 Å simulation box. During relaxations, geometries were optimized until residual forces were less than 0.04 eV/Å. Calculations were not run with spin polarization since our previous calculations suggested none of the studied systems have nonzero spin.^40^

The metal nanoparticles used an optimized double-ζ with two polarization orbitals (DZDP) basis set while an optimized double-ζ with single polarization (DZP) basis set was used for the adsorbates. The basis sets were optimized on the bulk metals and gas-phase molecules using SIESTA’s SIMPLEX minimization algorithm. The PBE functional^41^ was used with a mesh cutoff of 120 Ry, and Fermi smearing was used with an electronic temperature of 300 K. Norm-conserving pseudopotentials obtained from ABINIT’s Fritz-Haber-Institute pseudo database were used for all atoms. The orbital overlap matrix was calculated as implemented in the SISL Python package.^42^ The maximum overlap was determined by iterating over the orbital overlap matrix indices which correspond to the overlaps between the adsorbate and dopant. From this submatrix, the maximum value was extracted, providing a rough estimate of the propensity for charge transfer between the involved orbitals. The charge standard deviation σ_e_ was calculated by simply extracting the charge at all time points in the simulation and taking the standard deviation of all of these charges.

## Supporting Information Available

Computational methods, adsorption energies, additional N_2_ calculations, additional data on the electronic structure, simple tests of thermalization and the (111) facet, and additional calculations at varying heights (PDF)
