# Supplementary material for: Computational Discovery of Design Principles for Plasmon-Driven Bond Activation on Alloy Antenna Reactors
Source: ACS Nano. 2025 Mar 7;19(10):9860–7. doi: 10.1021/acsnano.4c13602 (PMC11924337; doi:10.1021/acsnano.4c13602)
Supplement: Supplementary file 1 — nn4c13602_si_001.pdf [file nn4c13602_si_001.pdf]

Supporting Information for "Computational discovery of design principles for plasmon-driven bond activation on alloy antenna reactors"

Connor J. Herring, Matthew M. Montemore*

Department of Chemical and Biomolecular Engineering, Tulane University, New Orleans LA 70115, USA

Email: mmontemore@tulane.edu

S1 Adsorption Energies

Table 1 shows the N_2 adsorption energy in the side-on (N_2 parallel to surface) and end-on (N_2 perpendicular to surface) orientation. N_2 is more strongly adsorbed in the end-on orientation for all alloys, and the two configurations are nearly isoenergetic for pure Cu.

Table 1: N_2 adsorption energies for both molecular orientations, side-on and end-on.

	N_2 adsorption energy (eV)	
Nanoparticle	Side-on	End-on
Cu	-0.10	-0.08
Ag_1Cu	-0.08	-0.09
Mo_1Cu	-1.00	-1.67
Re_1Cu	-1.03	-1.86
Ru_1Cu	-0.60	-1.30
Ti_1Cu	-1.13	-1.31

Figure S1 shows the relationship between the adsorption energy and bond activation for all molecules. H_2O shows no correlation, N_2 and CH_4 show some negative correlation, and CO_2

shows a positive relationship. Overall the adsorption strength does not appear to be solely responsible for or predictive of bond activation.

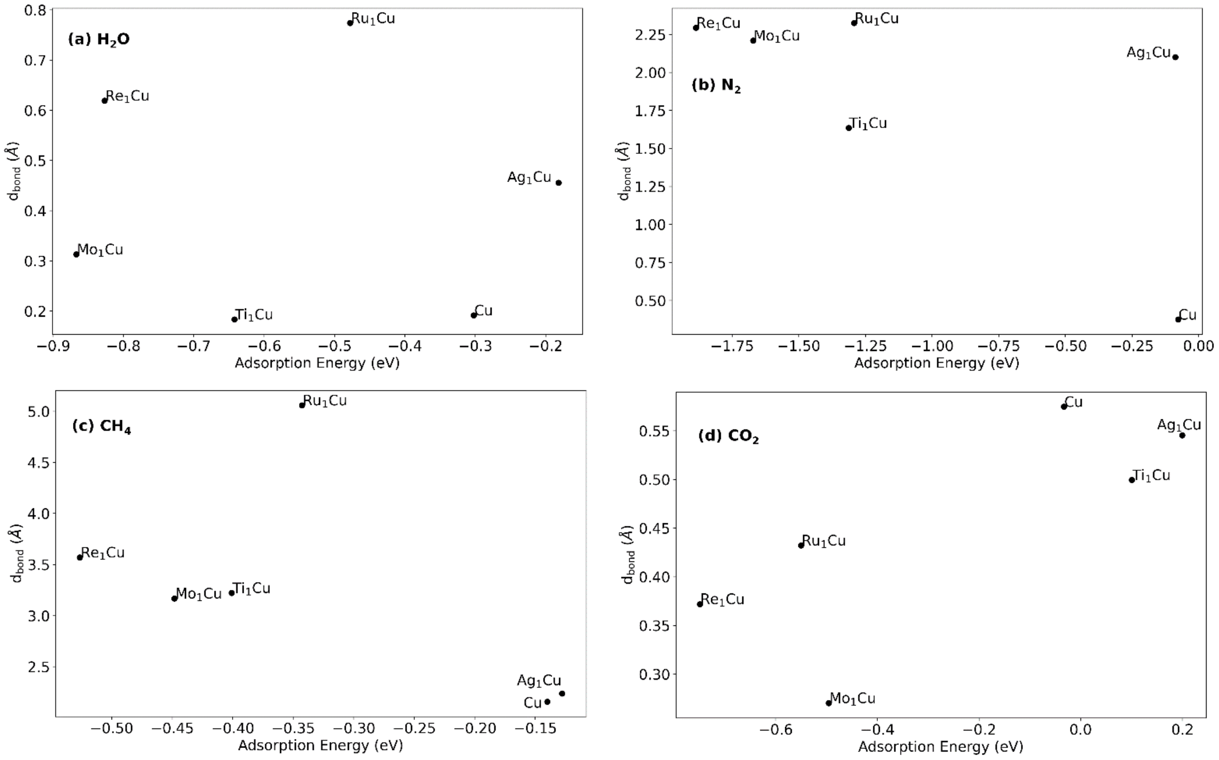

Figure S1: The bond length change for the most-activated bond (d_{bond}) vs. the adsorption energy for all molecules: (a) H_2O, (b) N_2, (c), CH_4, (d) CO_2.

S2 Additional N_2 Calculations

In addition to the calculations shown in the main text, nonadiabatic calculations were run with N_2 in the side-on geometry (Figure S2). In the side-on orientation all structures except for Ag_1Cu showed enhanced N_2 dissociation relative to the end-on adsorption geometries (Figure S2). We also performed calculations in the original end-on geometry with atomic positions fixed (Figure S3). The standard deviation of the N_2 charge change when atomic positions were fixed was found to correlate reasonably well with the N_2 bond length change when there were no constraints (Figure S4), indicating that the charge transfer was causing the dissociation rather than vice versa. Further

calculations include the charge density difference for N_2 in the end-on geometry (Figure S5) and the "critical" electric field value needed to induce dissociation (Figure S6).

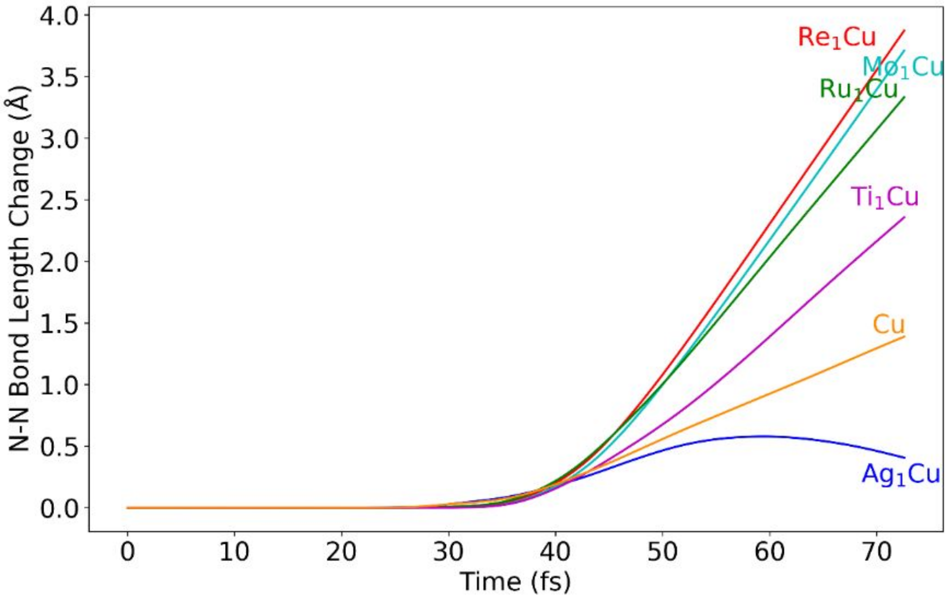

Figure S2: N_2 bond length change over time when N_2 is adsorbed in a side-on orientation and an electric field with a maximum amplitude of 0.1 Ry/Bohr/e is applied in the x, y, and z directions.

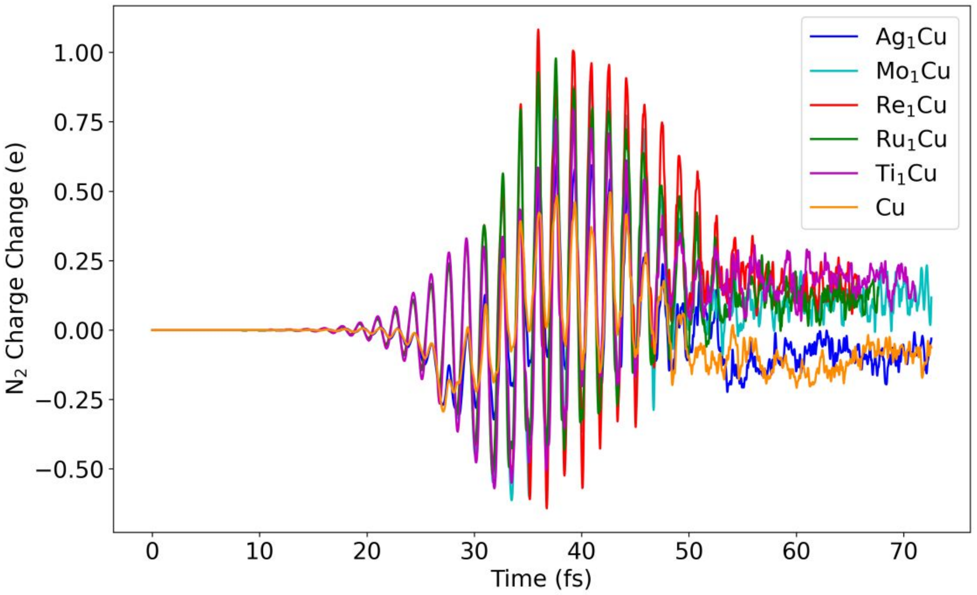

Figure S3: The charge change for N_2 (e) over time when atomic positions are fixed, and the system is subjected to the 0.1 Ry/Bohr/e electric field in all three directions.

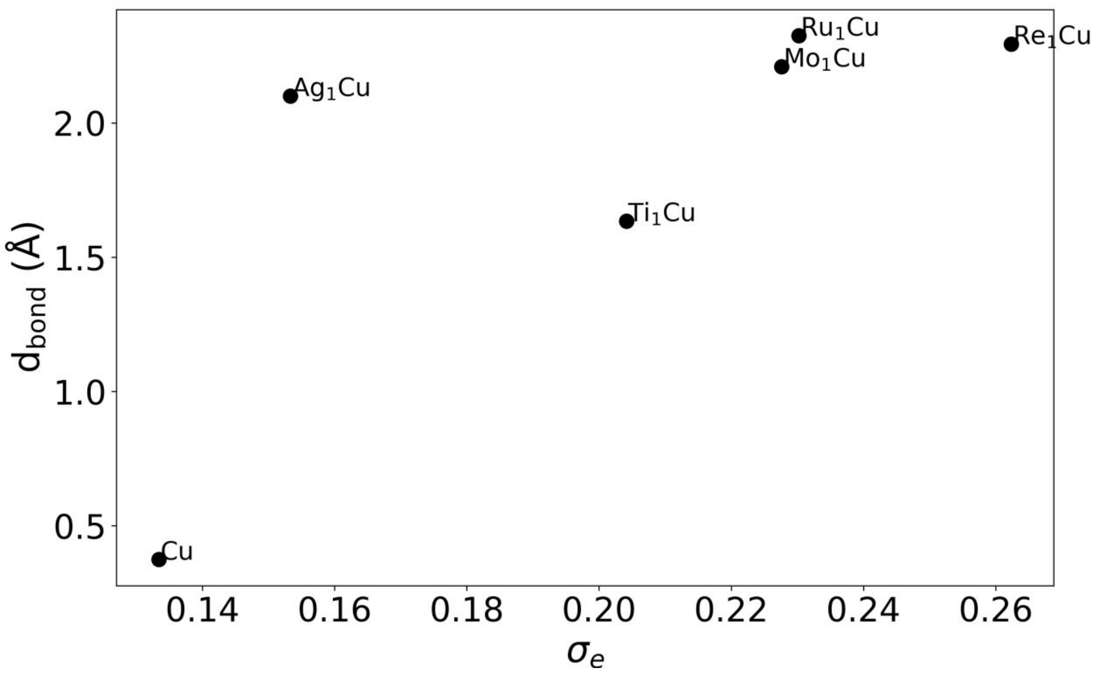

Figure S4: The value of d_{bond} when there are no constraints on atomic positions is plotted against the standard deviation in charge change, σ_e, on N_2 when atomic positions are fixed. The overall trend reported in the main text is largely the same.

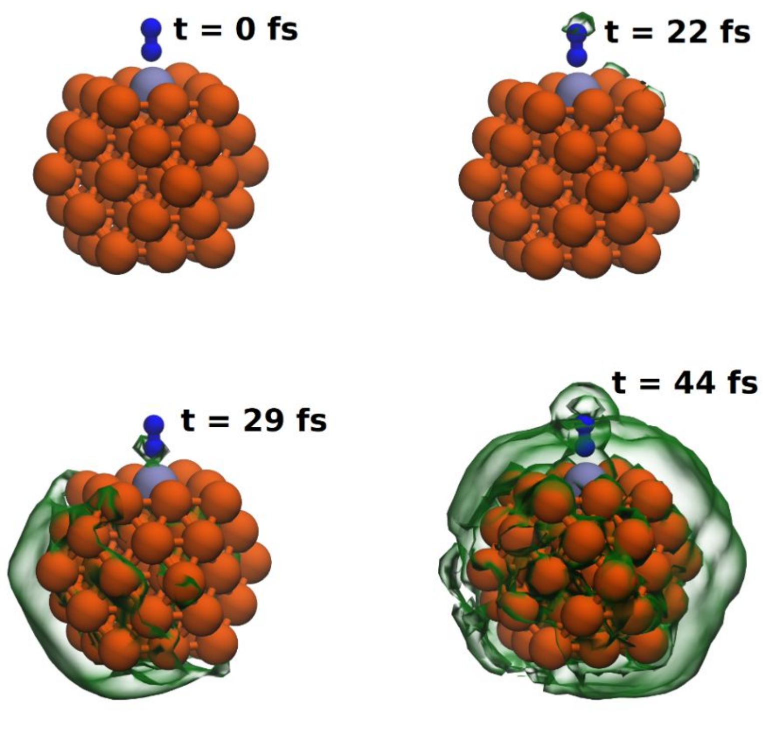

Figure S5: N_2 charge density difference on the Ru_1Cu nanoparticle at four time points as the electric field is applied and atomic positions are fixed. The initial excitation is located on the N atom farther from the surface.

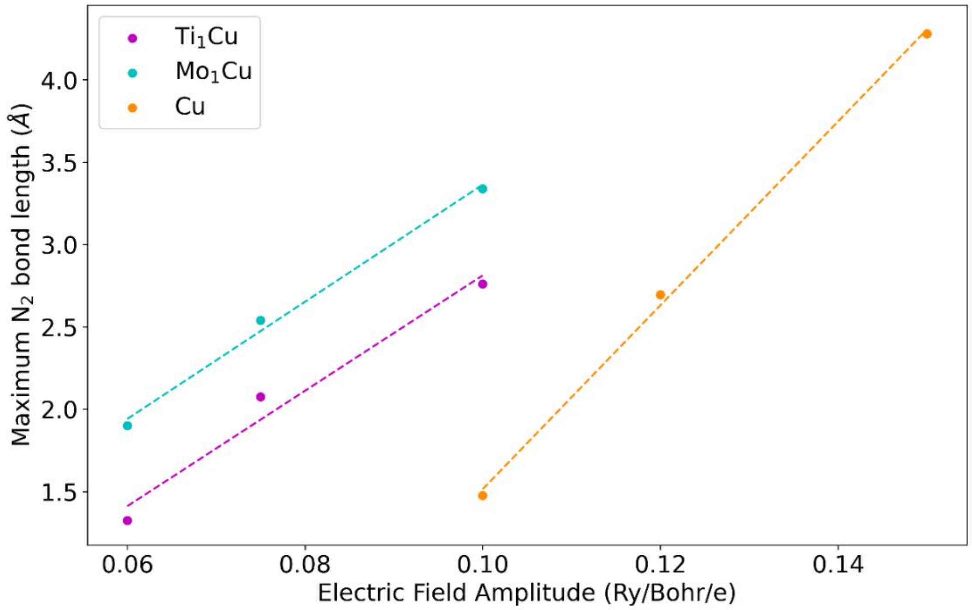

Figure S6: The external electric field amplitude (applied in the x, y, z) directions is plotted versus the maximum N_2 bond length reached for Mo_1Cu, Ti_1Cu, and pure Cu. Only the three points nearest the threshold for activation are shown. Using the lines of best fit the "critical" electric field amplitude (the value required for N_2 to reach its transition state bond length of 1.9 Å) was interpolated and found to be approximately 0.0603, 0.0739, 0.107 Ry/Bohr/e for Mo_1Cu, Ti_1Cu, and Cu, respectively.

S3 Charge Dynamics for all Molecules

As mentioned in the main text, Hirshfeld charge partitioning was used to find the charge on each atom over time. These atomic charges were then summed over each atom in a given molecule at each time step. Figure S7 shows the molecular charge change (relative to the molecule's initial charge) and Figure S8 shows the net molecular charge at every timepoint. Most molecules show relatively little charge change between the beginning and end of the simulation while CO_2 shows two distinct groupings based on initial chemical activation.

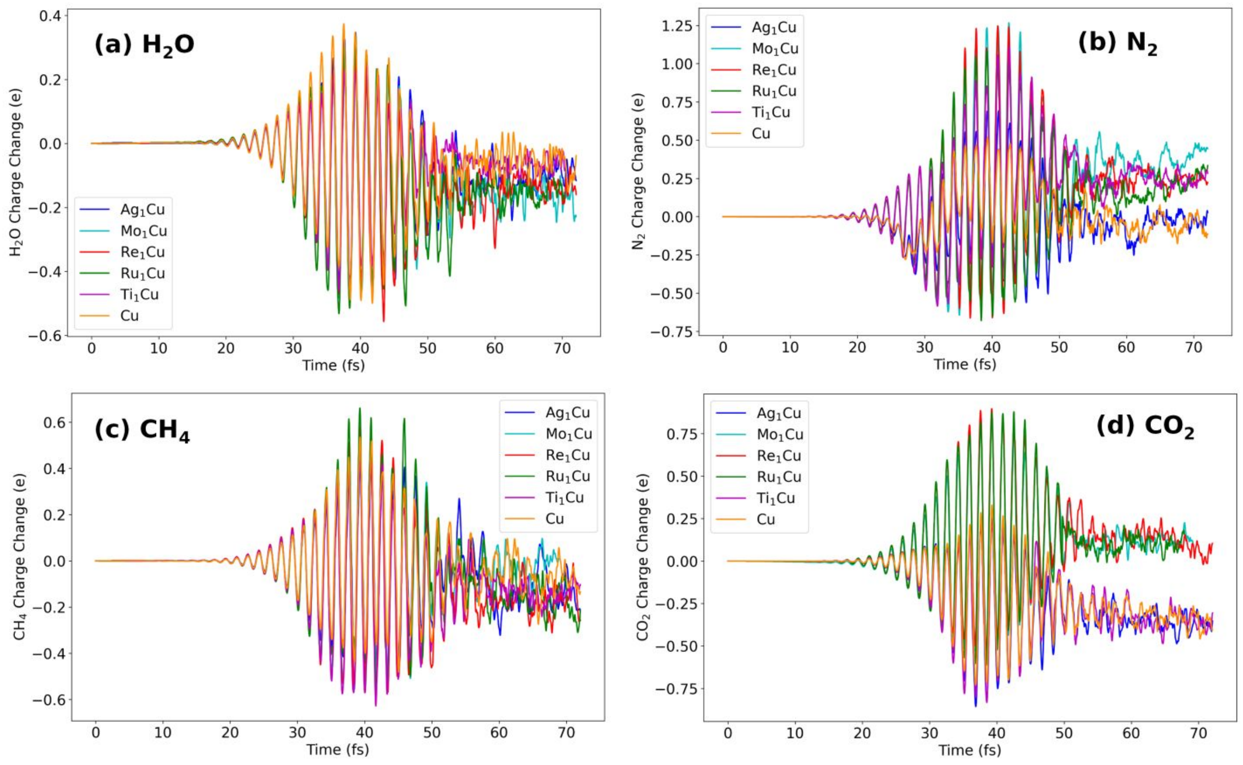

Figure S7: The molecular charge change (relative to initial molecular charge) over time for all four molecules: (a) H_2O, (b) N_2, (c), CH_4, (d) CO_2.

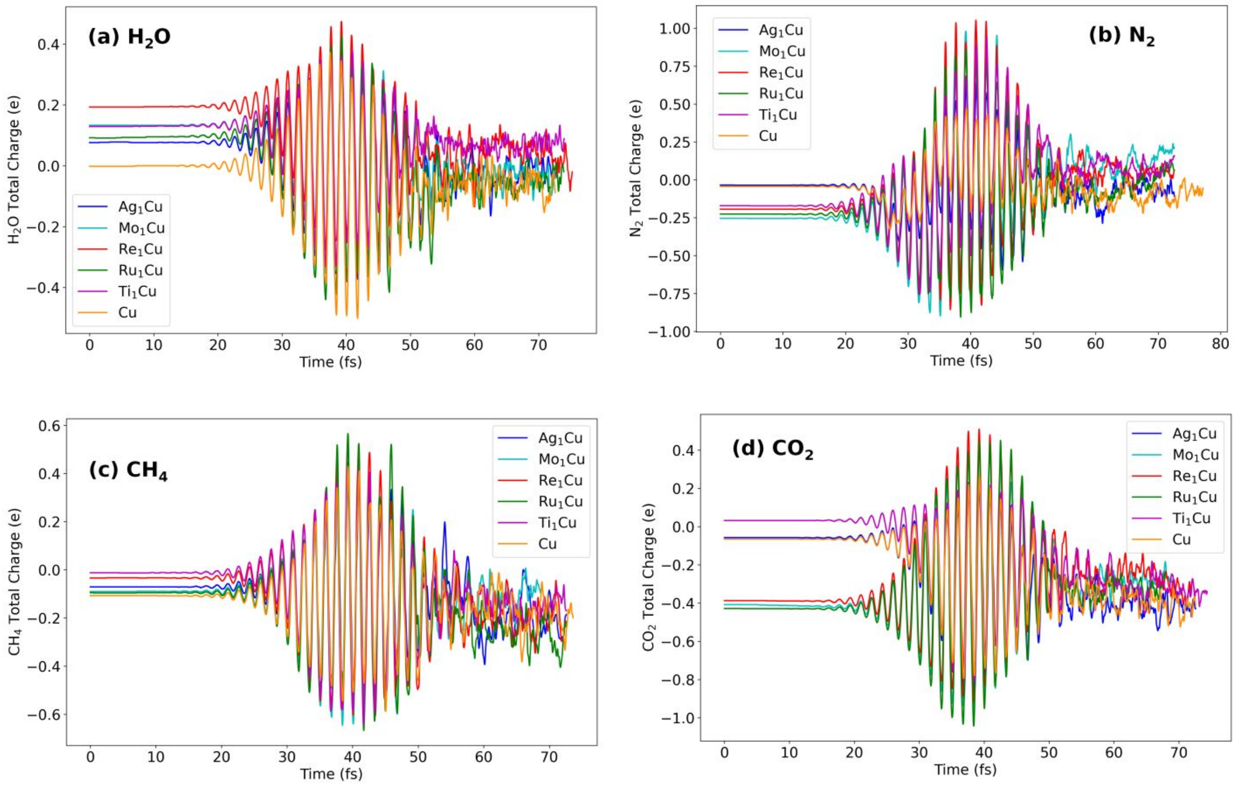

Figure S8: The total net charge is plotted for all four molecules: (a) H_2O, (b) N_2, (c), CH_4, (d) CO_2.

S4 Hamiltonian Matrix

Similar to the orbital overlap matrix discussed in the main text, the maximum value from the Hamiltonian matrix was extracted and plotted against the degree of bond activation d_{bond}. These results show that the Hamiltonian matrix corresponds reasonably well to bond activation and may be an alternative simple descriptor for screening plasmonic metal structures.

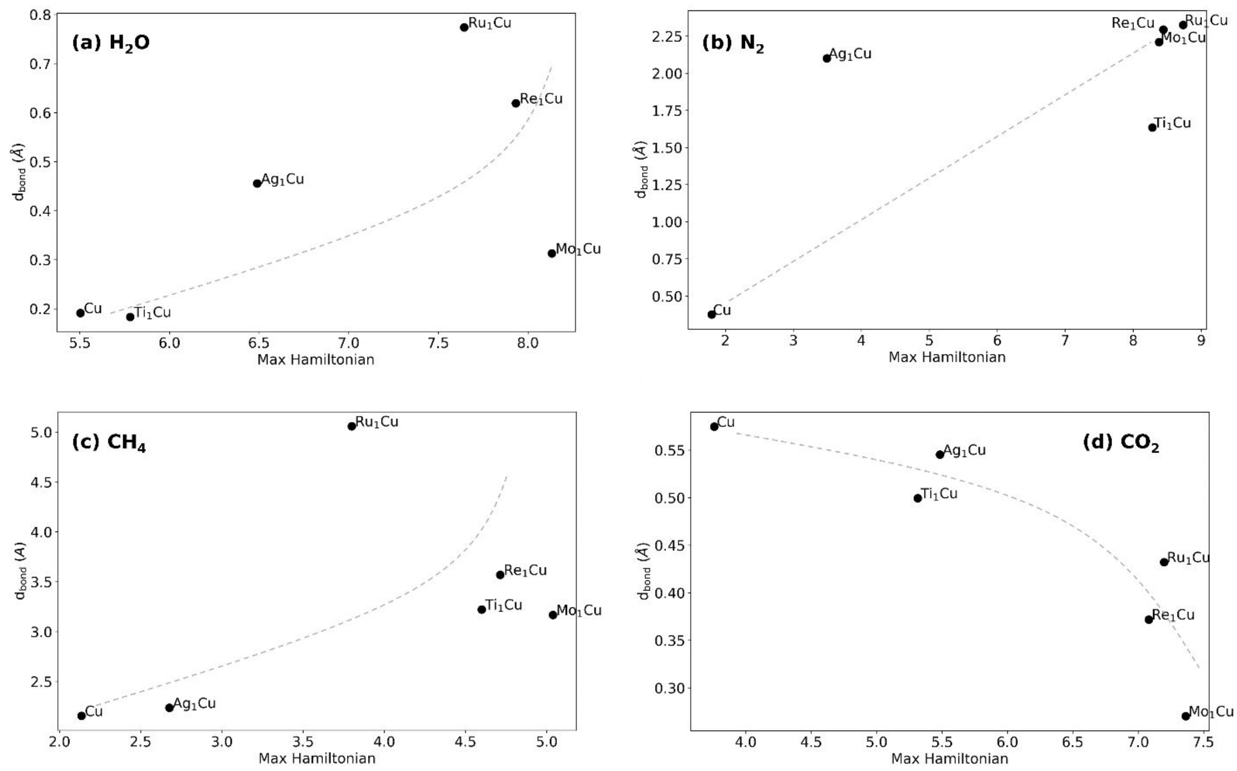

Figure S9: The the degree of bond activation d_{bond} is plotted against the maximum Hamiltonian matrix value between the molecule and the dopant atom. Lines have been added to guide the eye to the overall trends but are not lines of best fit.

S5 CO_2 PDOS

As discussed in the main text, CO_2 undergoes chemical activation upon adsorption on the Ru, Re, and Mo alloys. This leads to a decrease in bond angle and an increase in the energies of the

unoccupied states in CO_2. In addition to the CO_2 states (plotted in red and scaled by a factor of 10) the nanoparticle states are plotted in grey for each structure.

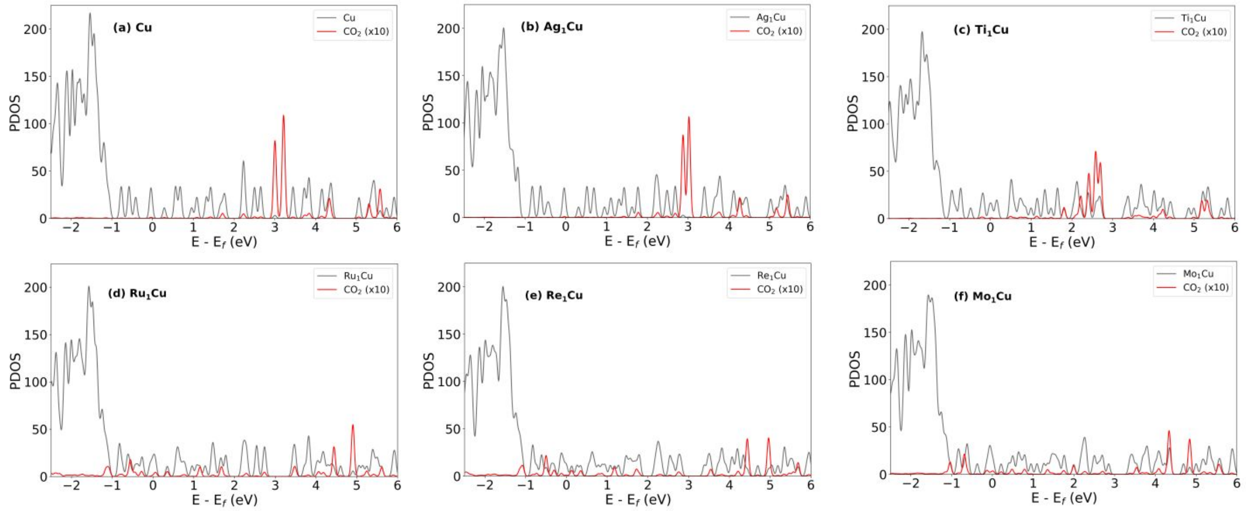

Figure S10: Projected density of states for the nanoparticle (grey) and CO_2 (red) is plotted for each system: (a) Cu, (b) Ag_1Cu, (c) Ti_1Cu, (d) Ru_1Cu, (e) Re_1Cu, (f) Mo_1Cu. The values for CO_2 are scaled by a factor of 10 for visual clarity. CO_2 unoccupied states shift to higher energies for the chemically activated cases (d-f).

S6 Cu and Ru_1Cu Charge Density Difference

We performed charge density difference calculations for two bare nanoparticles (pure Cu and Ru_1Cu). These show the difference in charge density between ground state and nonadiabatic electron densities along the nonadiabatic trajectory. Only three snapshots are shown corresponding to the early stages of the excitation as beyond this point differences between the two structures appear to be negligible.

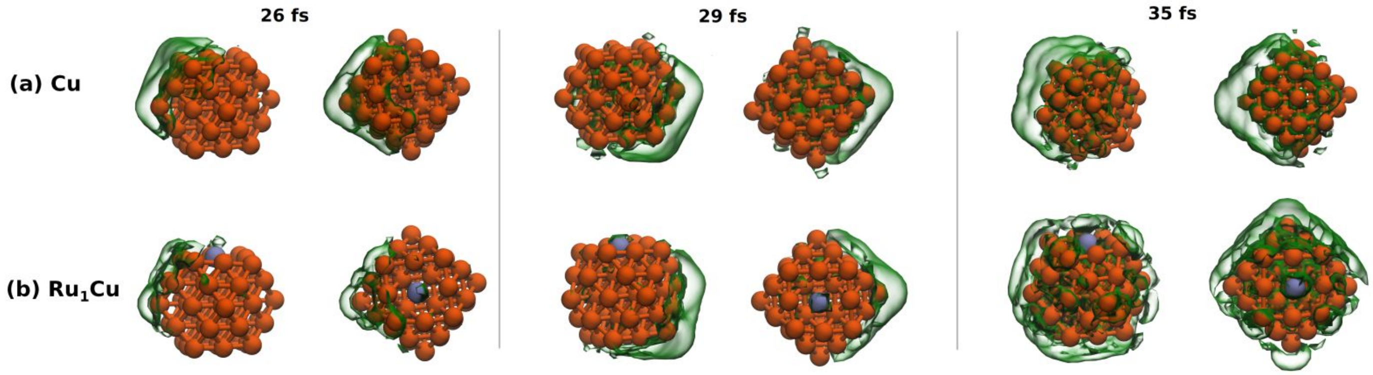

Figure S11: The charge density difference between the nonadiabatic simulation and the ground state at the same geometry at three time points is shown for (a) Cu and (b) Ru_1Cu, with an isosurface value of 10^{-3} e/Å. Ru localizes slightly more charge density on the surface but the overall shape of the plasmon is unaffected by the dopant atom.

S7 Experimental Comparisons

In addition to the Ru_1Cu and pure Cu simulations with CH_4 reported in the main text, five other structures were simulated for the purpose of comparing RT-TDDFT to experiment. These included a 55 atom Au nanoparticle as compared to a 9 atom Pt cluster on a Au_{55} nanoparticle (Pt_9Au_{55}), a 55 atom Ru nanoparticle as compared to a 9 atom Ru cluster on Pd_{55} (Ru_9Pd_{55}), as well as a Rh_1Cu nanoparticle as compared to pure Cu. The structures and molecules were relaxed prior to applying the electric field. The bond length change for the adsorbates is shown in Figures S12-S15 and summarized in Table S2. Simulations were run with the applied electric field frequency using an amplitude of 1.54 V/Å for CH_4 and 2.57 V/Å for N_2. The bond length change refers to the maximum bond length reached between 0 and 55 fs relative to the initial bond length. The agreement between RT-TDDFT predictions and experimental findings supports the use of our methodology as a metric for estimating photocatalytic activity trends.

Table S2: RT-TDDFT calculated bond length changes for CH_4 or N_2 on various pure metal nanoparticles and antenna reactor complexes. For CH_4 the bond length change refers to the most activated C-H bond while for N_2 this refers to the N-N bond. All antenna reactors show significant enhancement in molecular bond activation, consistent with experimental findings of improved conversion of each molecule.

Nanoparticle	Molecule	Bond Length Change (Å)	Electric Field Frequency (nm)	Reference
Cu	CH_4	0.77	500	1
Ru_1Cu	CH_4	2.13	500	1
Au	CH_4	0.37	520	2
Pt_9Au_{55}	CH_4	4.43	520	2
Ru	N_2	0.93	500	3
Ru_9Pd_{55}	N_2	2.11	500	3
Rh_1Cu	CH_4	1.86	500	4
Cu	CH_4	0.77	500	4

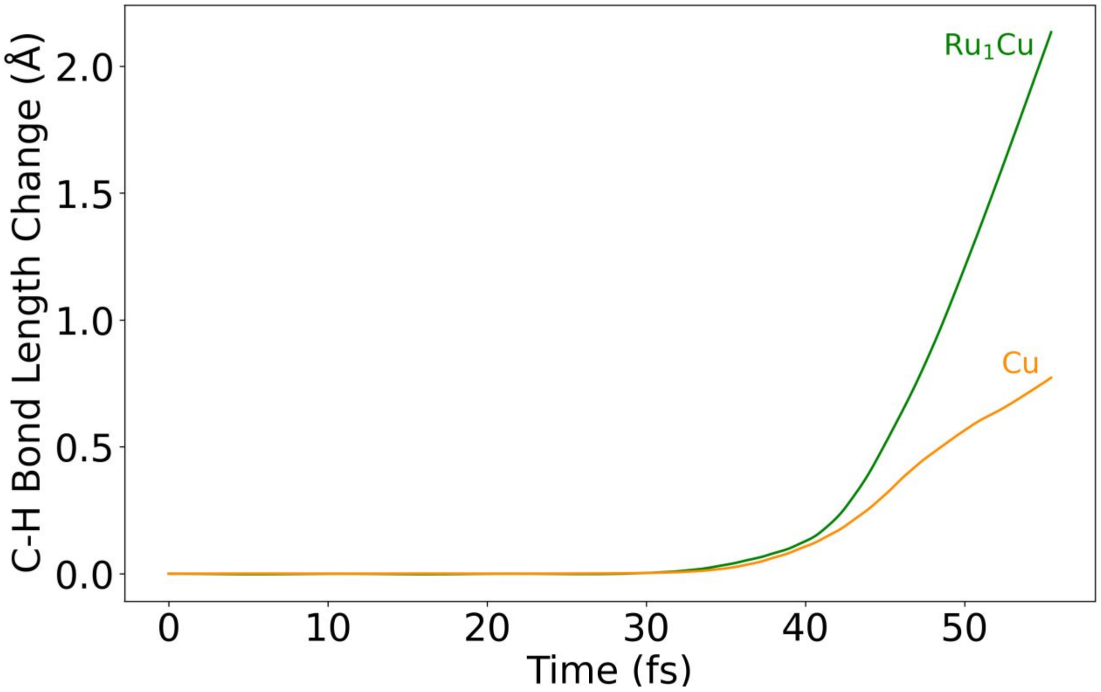

Figure S12: The largest C-H bond length change in CH_4 is plotted when adsorbed on the Ru_1Cu and Cu nanoparticles and an electric field is applied. The Ru_1Cu antenna reactor shows a significant enhancement in C-H bond activation.

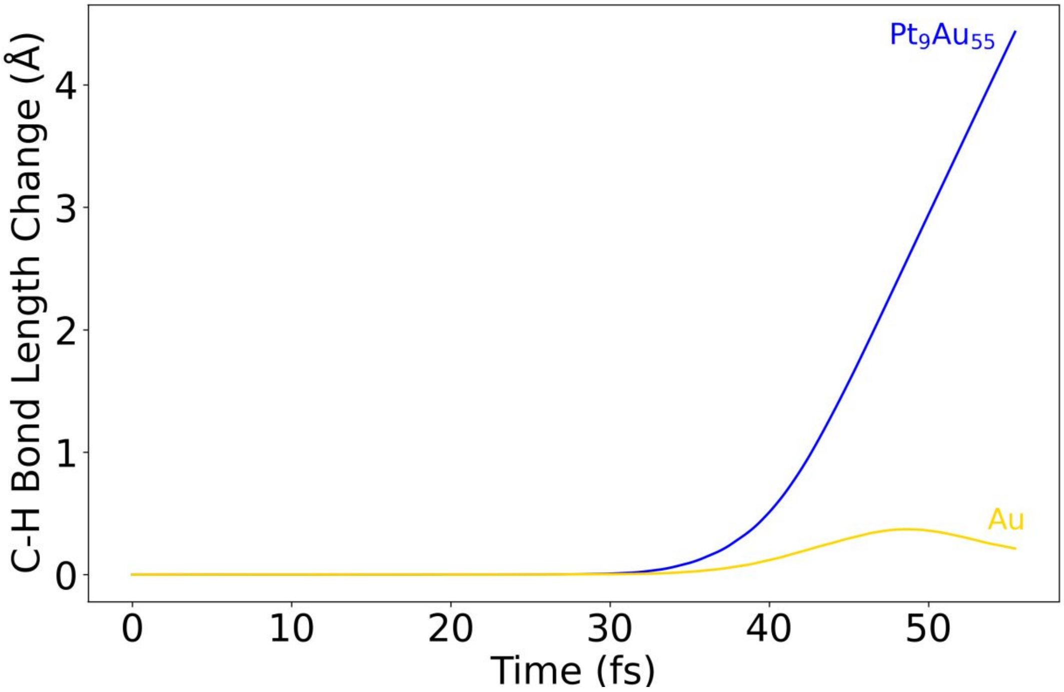

Figure S13: The largest C-H bond length change in CH_4 is plotted when adsorbed on the Pt_9Au_{55} and Au nanoparticles and an electric field is applied. The Pt_9Au_{55} antenna reactor shows a significant enhancement in C-H bond activation.

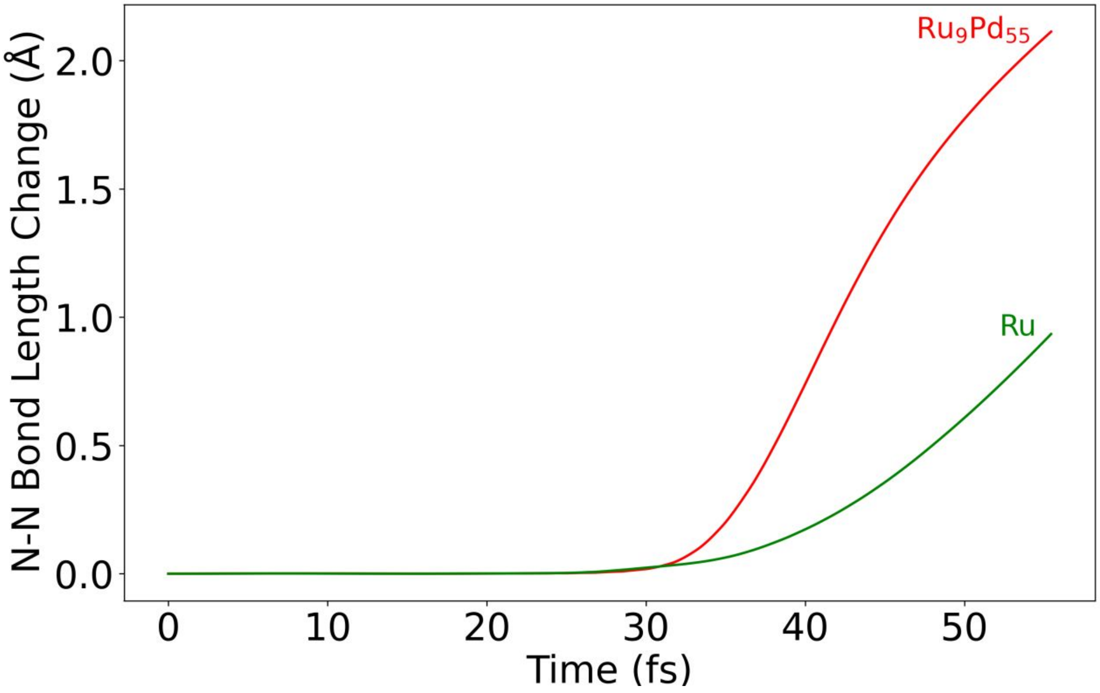

Figure S14: The N-N bond length change is plotted when adsorbed on the Ru_9Pd_{55} and Ru nanoparticles and an electric field is applied. The Ru_9Pd_{55} antenna reactor shows a significant enhancement in N-N bond activation.

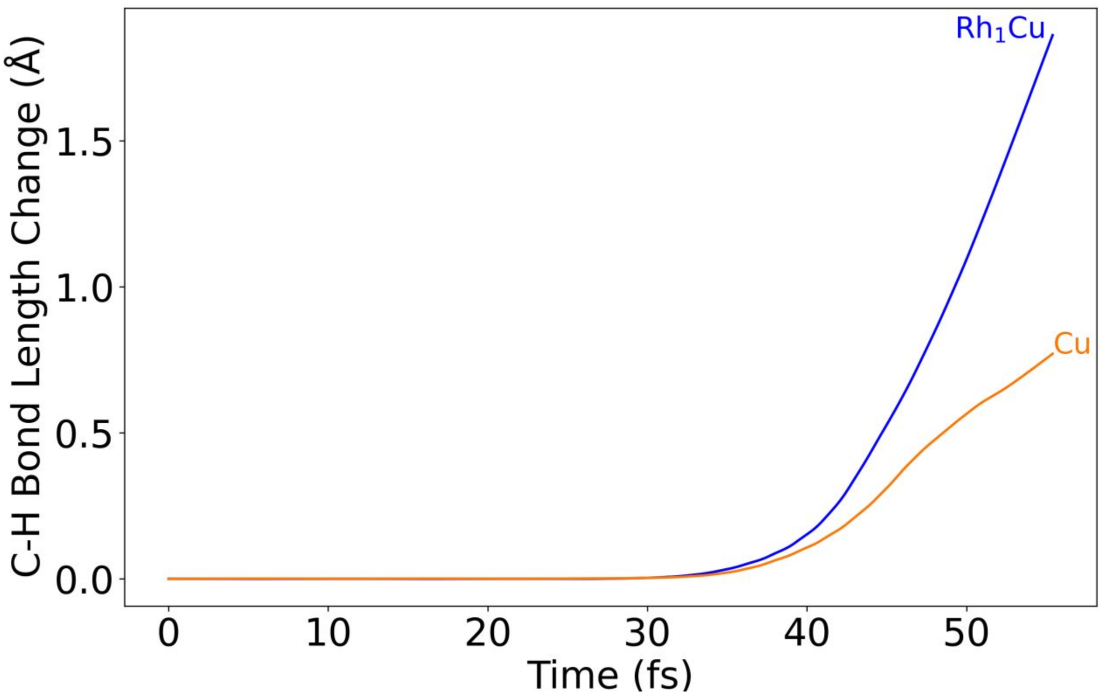

Figure S15: The largest C-H bond length change in CH_4 is plotted when adsorbed on the Rh_1Cu and Cu nanoparticles and an electric field is applied. The Rh_1Cu antenna reactor shows a significant enhancement in C-H bond activation.

S8 Alternate Facet Calculations

Three additional calculations were run using CH_4 adsorbed on the step edge of a (111) facet of Ru_1Cu, Ti_1Cu and pure Cu. A larger nanoparticle with 79 atoms was used, as the 55 atom nanoparticles used in the rest of our calculations have very small (111) facets. These calculations used a maximum amplitude of 0.07 Ry/Bohr/e, which we found to give clear trends across these nanoparticles. While the dynamics were quantitatively different between the (100) facet of the small nanoparticle and the (111) facet of the larger nanoparticle, the Ru_1Cu and Ti_1Cu alloys still exhibited an enhancement compared to pure Cu, suggesting that the trends across compositions is

likely to be similar across different facets and nanoparticle sizes.

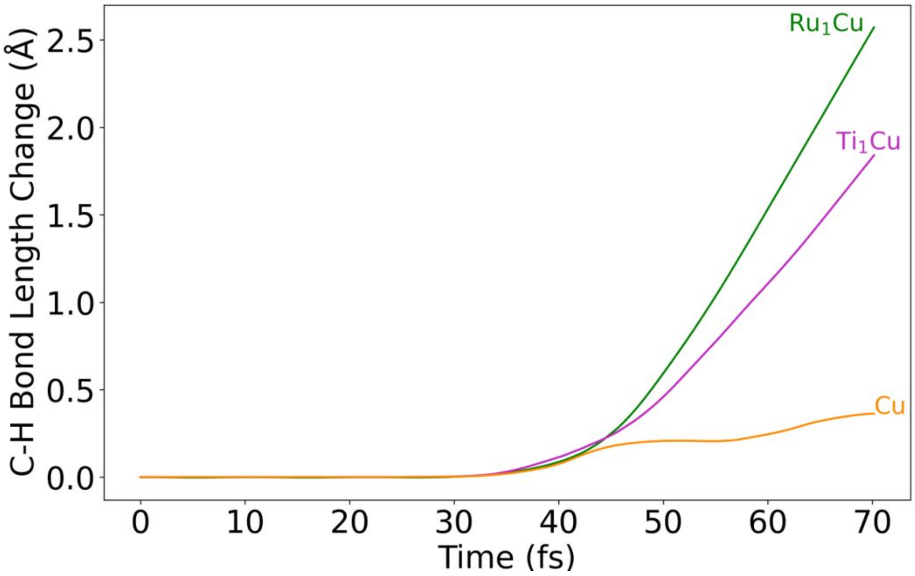

S16: The largest C-H bond length change in CH_4 is plotted when adsorbed on the step edge of a (111) facet on Ru_1Cu, Ti_1Cu, and Cu nanoparticles and an electric field is applied. The Ru_1Cu and Ti_1Cu antenna reactors still show a significant enhancement in C-H bond activation relative to pure Cu.

S9 Thermal Effects

To thermalize CH_4 on Cu and Ru_1Cu, we ran a short ground-state MD calculation with an initial temperature of 600 K. We then took the output geometry and velocities (corresponding to a temperature of ~400 K) and ran the RT-TDDFT calculations with the external field. The most activated C-H bond was further activated when the initial temperature was used on both Ru_1Cu and Cu (Figure S17). This suggests that thermal effects may contribute to the dynamics but do not alter trends seen in the main text. Notably, the C-H bond on Cu at 400 K was still not as activated as on Ru_1Cu with no initial temperature, suggesting that the photochemical enhancement by the antenna reactor contributes more than thermal effects, at least at for these conditions.

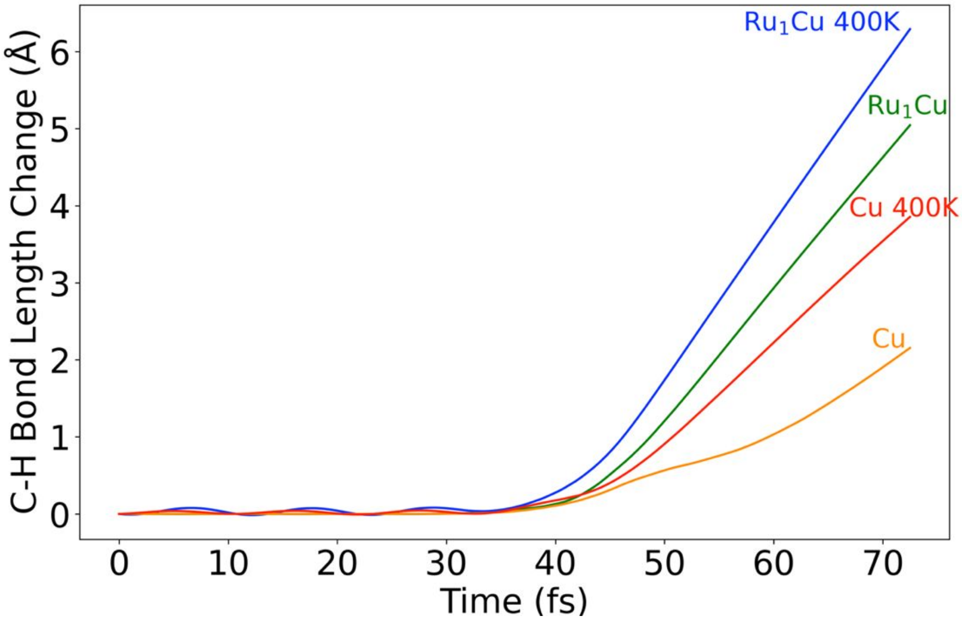

Figure S17: The C-H bond change is shown for the most activated bond in CH_4 when adsorbed on Ru_1Cu and pure Cu with initial velocities corresponding to ~400 K, as well as the same nanoparticles at 0 K.

S10: N_2 on Ru_1Cu at Variable Heights

In addition to the relaxed N_2 height and the distance of ~3.1 Å, we ran two more calculations for N_2 adsorbed on Ru_1Cu to see how this may impact charge transfer and the subsequent molecular dissociation. Our results show that moving the molecule farther from the nanoparticle surface (3.5 and 4.1 Å) decreases the transient charge oscillations and the degree of bond activation

(Figure S18), following generally the same trend as the original alloy data.

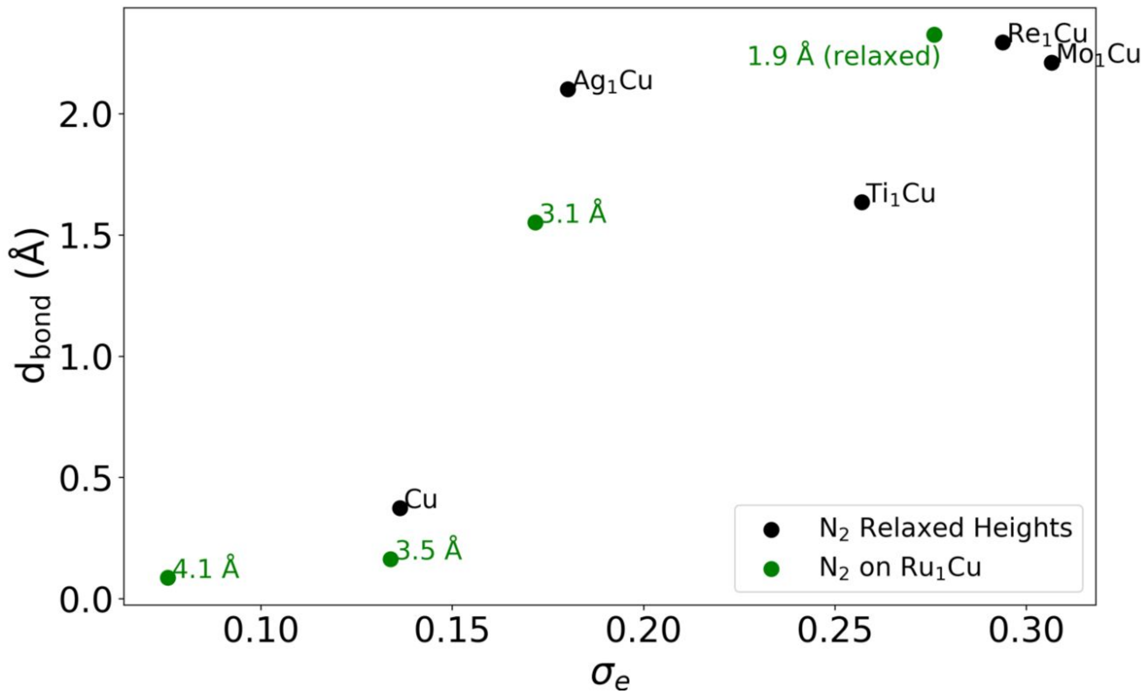

Figure S18. N-N bond activation vs. the charge standard deviation for N_2 on all of the nanoparticles at the relaxed height (same as Figure 2d) and for N_2/Ru_1Cu at varying heights. All of these systems follow roughly the same trend.

References

(1) Zhou, L.; Martirez, J. M. P.; Finzel, J.; Zhang, C.; Swearer, D. F.; Tian, S.; Robatjazi, H.; Lou, M.; Dong, L.; Henderson, L.; Christopher, P.; Carter, E. A.; Nordlander, P.; Halas, N. J. Light-Driven Methane Dry Reforming with Single Atomic Site Antenna-Reactor Plasmonic Photocatalysts. *Nat. Energy* **2020**, *5* (1), 61–70. https://doi.org/10.1038/s41560-019-0517-9.

(2) Song, H.; Meng, X.; Dao, T. D.; Zhou, W.; Liu, H.; Shi, L.; Zhang, H.; Nagao, T.; Kako, T.; Ye, J. Light-Enhanced Carbon Dioxide Activation and Conversion by Effective Plasmonic Coupling Effect of Pt and Au Nanoparticles. *ACS Appl. Mater. Interfaces* **2018**, *10* (1), 408–416. https://doi.org/10.1021/acsami.7b13043.

(3) Yang, Y.; Jia, H.; Su, S.; Zhang, Y.; Zhao, M.; Li, J.; Ruan, Q.; Zhang, C. Y. A Pd-Based Plasmonic Photocatalyst for Nitrogen Fixation through an Antenna-Reactor Mechanism. *Chem. Sci.* **2023**, *14* (39), 10953–10961. https://doi.org/10.1039/d3sc02862c.
